# Neoplastic Incidentalomas in the Emergency Department: Incidence, Imaging Type, Location, and Clinical Relevance

**DOI:** 10.5811/westjem.48928

**Published:** 2026-06-28

**Authors:** Carmine Nasta, Romeo Morelli, Antonia Ida Facciuto, Francesca Palumbo, Martina Finelli, Vincenzo Brunelli, Mario Puoti, Alfredo Palumbo, Antonio Voza, Giuseppe Paolisso, Mauro Giordano

**Affiliations:** * Università degli Studi della Campania Luigi Vanvitelli, Naples, Department of Advanced Medical and Surgical Sciences, Department of Emergency Medicine, Marcianise, Italy; † Istituto di Ricovero e Cura a Carattere Scientifico Humanitas Research Hospital, Department of Biomedical Sciences, Milan, Italy

## Abstract

**Background:**

Incidentalomas—unexpected neoplastic lesions discovered during imaging for unrelated complaints—represent a growing clinical and ethical challenge in emergency medicine. Their detection has increased due to widespread use of advanced imaging, particularly in patients who may otherwise escape thorough diagnostic evaluation.

**Objective:**

We sought to determine the frequency, anatomical distribution, and clinical management gaps of neoplastic incidentalomas observed in patients presenting with non-specific, low-severity complaints at the emergency department (ED). (In this paper, neoplastic incidentalomas include both lesions discovered incidentally during imaging for unrelated conditions and newly diagnosed malignancies plausibly related to the patient’s presenting symptoms,)

**Methods:**

Data collection covered the period January 1–December 31, 2023. We conducted a retrospective observational study of all adult patients presenting to the ED of P.O. Anastasia Guerriero University Hospital in Marcianise, Italy. Incidentalomas were identified through discharge summaries and classified by imaging modality, anatomical site, and triage code. We analyzed data using descriptive and inferential statistics.

**Results:**

Among 35,081 ED visits, 53 neoplastic incidentalomas were identified (1.52 per 1,000). The majority occurred in females (69%) and elderly (> 65 years) patients (61%), who often presented with minor complaints such as abdominal pain (38%) or anemia (15%). Most incidentalomas were gastrointestinal (31%) or pulmonary (23%) and primarily detected via computed tomography (51%). Notably, 100% of cases were triaged as non-urgent. Based on established guidelines, up to 20% of cases may represent potential overdiagnosis. The next most common modalities of detection were ultrasound (28%) and chest radiograph (22%).

**Conclusion:**

Neoplastic incidentalomas are not rare in the ED setting, even among patients who present with low-severity symptoms. Our findings underscore the importance of structured follow-up protocols and integration of guideline-based reporting to avoid diagnostic delays, overdiagnosis, and unnecessary interventions. Emergency physicians should maintain diagnostic vigilance and consider incidental findings in clinical workflows.

## INTRODUCTION

The term incidentaloma refers to an unexpected mass or lesion detected during diagnostic studies performed for unrelated clinical indications. First coined in the early 1980s in reference to adrenal masses,^1^ the term now encompasses a broad range of anatomical sites and clinical implications. With the increased use of advanced imaging in emergency departments (ED), the detection of incidentalomas has become more frequent, raising critical questions regarding diagnosis, follow-up, and patient communication.^2^ This study investigates the prevalence and clinical profiles of neoplastic incidentalomas in a busy Italian ED, focusing on patients admitted for low-severity complaints and highlighting the implications for emergency clinical practice. Neoplastic incidentalomas encompassed two distinct clinical scenarios: 1) truly incidental lesions discovered during imaging for unrelated conditions; and 2) newly diagnosed malignancies that may have explained the patient’s presenting symptoms.

## MATERIALS AND METHODS

### Study Design, Setting, and Population

This was a retrospective, observational, cohort study conducted in the ED of the University Hospital in Marcianise, Italy. All adult patients (>18 years of age) who presented to the ED from January–December 31, 2023 were eligible to participate in the study. Exclusion criteria were as follows: patients who were < 18 years of age; and those who had incomplete records.

### Data Collection

We screened ED) discharge summaries for mention of incidental neoplastic findings. Data collected included demographics, reason for ED visit, triage code, type of imaging performed, and anatomical site of the incidentaloma. The review of ED discharge summaries was conducted January–March 2024 by two independent reviewers trained in clinical epidemiology. Discrepancies were resolved by consensus with a third senior investigator. We operationally defined neoplastic incidentalomas as radiologically confirmed solid lesions suggestive of malignancy, discovered during diagnostic imaging performed for symptoms not always directly attributable to cancer. For example, a pulmonary nodule identified during imaging for chest pain without signs of infection or hemoptysis was considered incidental. Additional examples include the following: gastrointestinal (GI) lesions (e.g., colonic masses identified on computed tomography [CT] performed for abdominal pain); breast findings (e.g., lesions detected incidentally after imaging for trauma); ovarian masses (e.g., adnexal tumors revealed on ultrasound for abdominal bloating); cerebral lesions (e.g., tumors discovered following imaging for seizures); and renal findings (e.g., masses detected on CT performed for anemia or hematuria).

For this study, we included both truly incidental neoplastic findings, defined as lesions detected during imaging performed for unrelated complaints, such as a renal mass identified on CT abdomen for suspected appendicitis, and newly diagnosed malignancies that could plausibly be related to the patient’s presenting symptoms, such as a colonic mass in a patient with abdominal pain and weight loss. Both categories were reviewed and reported together under the umbrella of “neoplastic incidentalomas,” while acknowledging their distinct clinical implications. We used descriptive statistics to summarize patient demographics and incidentaloma characteristics. Categorical variables were reported as frequencies and percentages. Continuous variables were expressed as mean ± standard deviation or median with interquartile range, as appropriate. We applied chi-square or Fisher exact tests to compare categorical data across subgroups (eg, sex, age group). A *P*-value < .05 was considered statistically significant. We analyzed data using IBM SPSS Statistics for Windows, v28.0 (IBM Corporation, Armonk, NY). All data were anonymized; ethics committee approval was not required for retrospective analysis of administrative data.

**Population Health Research Capsule**

What do we already know about this issue?

*Incidental neoplastic findings are being detected with increasing frequency due to the widespread use of advanced imaging in emergency departments*.

What was the research question?

*What is the incidence, distribution, and clinical significance of neoplastic incidentalomas among ED patients?*

What was the major finding of the study?

*In 35,081 ED visits, 53 neoplastic incidentalomas were identified (1.52%), mainly in elderly females, with computed tomography the most common imaging modality (51% [P < .001])*.

How does this improve population health?

*Our findings highlight the concealed oncologic risk in low-severity presentations and promotes adherence to evidence-based follow-up pathways to reduce diagnostic latency and overdiagnosis*.

The methodology followed established best practices for retrospective chart review studies in emergency medicine. In particular, the data abstraction process adhered to criteria proposed by Worster and Bledsoe, Gilbert and Lowenstein, and Kaji and Schriger,^4^,^5^ which emphasize independent review, predefined data fields, and reconciliation of discrepancies to enhance data reliability. While we adhered to elements from Worster and Bledsoe including use of predefined abstraction fields, independent chart reviewers, and consensus-based discrepancy resolution, not all their methodological recommendations were followed. Specifically, no formal abstractor training was conducted, inter-rater reliability was not quantified, and reviewers were not blinded to the study objectives. Additionally, the reporting of this observational study complies with the STROBE (Strengthening the Reporting of Observational Studies in Epidemiology) guidelines, to ensure transparency and reproducibility in study design, analysis, and interpretation.^5^,^6^

## RESULTS

In 35,081 ED visits, 53 incidentalomas were identified, corresponding to an overall prevalence of 1.52 per 1,000 visits (95% CI, 1.12–1.98). Among patients ≥ 65 years of age, the prevalence increased to 2.47 per 1,000 (95% CI, 1.75–3.42). Incidentalomas were more frequent in women than in men (1.85 vs 1.07 per 1,000 visits, *P* = .09), and patients in the ≥ 65 year of age group were significantly more likely to have an incidentaloma compared to younger cohorts (*P* < .01). Most patients with incidentalomas were female (69%), while 31% were male. The majority were > 65 years of age (61%), with 31% between 40–65 and 8% between 18–40 years of age. At triage, 62% were classified as green (minor urgency) and 38% as blue (deferred urgency). ^(13)^ The most frequent presenting complaint was abdominal pain (38%), followed by severe anemia (15%) and constipation (7%); less common symptoms included seizures, hemoptysis, dyspnea, urinary tract infection, and breast mass.[Table t2-wjem-27-842]

Computed tomography was the most common imaging modality leading to detection (51%), followed by ultrasound (28%) and chest radiograph (22%). Incidentalomas detected on CT were more often suspicious for malignancy (78%) compared with those found on ultrasound (42%) or chest radiograph (31%) (*P* = .02). In terms of anatomical distribution, incidentalomas were most frequently GIl (31%) or pulmonary (23%), while 15% each involved the breast and ovaries. Cerebral and renal findings each accounted for 8%. Site-symptom associations showed that GI tumors were strongly linked to abdominal pain (82%), pulmonary lesions to non-specific complaints such as dyspnea or fatigue (61%), and breast lesions were exclusively detected in women presenting with unrelated issues such as trauma or dizziness. Regarding patient disposition, 72% were discharged directly from the ED, 21% required hospitalization, and 7% were transferred to other facilities.

**Figure 1:**
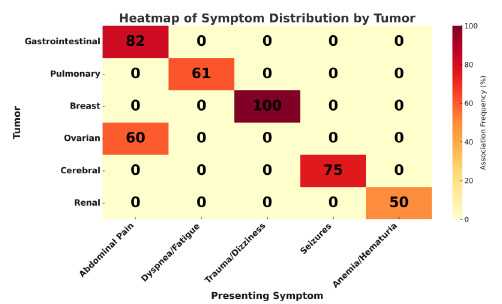
Heatmap of symptom distribution by tumor site in a study of the prevalence of incidentalomas detected in the emergency department.

The heatmap in Figure 1 illustrates the frequency of association (percentage) between presenting symptoms and anatomical sites of neoplastic incidentalomas detected in the ED. Gastrointestinal tumors were most frequently associated with abdominal pain (82%), while pulmonary incidentalomas correlated with dyspnea or fatigue (61%). Breast incidentalomas were detected in patients presenting with unrelated symptoms such as trauma or dizziness (100%). The intensity of color reflects the strength of association.

## DISCUSSION

This study provides single-center, real-world evidence from a high-volume ED and highlights under-recognized cancer risk in low-severity triage presentations. Neoplastic incidentalomas are not rare events in the ED, particularly among patients presenting with non-specific, low-severity complaints. An important nuance of our findings is that the category of neoplastic incidentalomas encompassed two distinct clinical scenarios: 1) truly incidental lesions discovered during imaging for unrelated conditions; and 2) newly diagnosed malignancies that may have explained the patient’s presenting symptoms. The latter group is particularly relevant in the emergency context, as these cases reflect situations where potentially serious oncologic disease initially manifested with vague or non-specific complaints and was only recognized because of imaging. This distinction highlights the need for careful clinical interpretation, since not all detected malignancies represent incidental or unrelated findings. In fact, all cases were identified in patients assigned green or blue triage codes,^7^ traditionally considered non-urgent. Our findings underscore a paradox: potentially serious oncologic conditions may initially present with vague symptoms and low-priority triage classifications, thus escaping early recognition unless incidental findings prompt further investigation. The predominance of GI and thoracic tumors aligns with previous data reported in literature.^8^,^9^

The widespread use of CT imaging played a crucial role in identification, reinforcing the need for standardized protocols. Observed sex and age trends support the importance of considering incidentalomas in older female populations. In the present study, we report a prevalence of 1.52 per 1,000, consistent with previous reports from high-volume EDs in North America and Western Europe, which estimate incidentaloma detection rates of 0.8–2.0 per 1,000 visits.^10^,^11^ Our findings regarding the predominance of GI and pulmonary lesions are in line with data previously reported in the literature.^10^,^11^ We report, for the first time, that in patients presenting with non-specific, low-severity pathologies at first ED visit there is a risk of underestimating the presence of neoplastic incidentalomas, particularly in patients with abdominal pain and anemia, as these were found in at least 50% of cases. In addition, these patients may be prematurely discharged without adequate follow-up planning, leading to delayed cancer diagnosis.[Table t3-wjem-27-842]

Our results suggest the need for structured documentation and handoff protocols for incidental radiologic findings, particularly in elderly female patients—a demographic disproportionately represented in our cohort. Furthermore, we need to be aware of ethical considerations regarding overdiagnosis. A significant proportion of the detected lesions may represent overdiagnosis, especially in asymptomatic elderly individuals with decreased life expectancy. Without clear follow-up protocols, incidentalomas may trigger cascade effects—unnecessary diagnostics, overtreatment, and patient anxiety—raising concerns about the balance between early detection and harm. Future prospective studies should incorporate validated patient-reported outcome measures to assess psychological and quality-of-life impacts.

### Recommendations for Clinical Practice

Based on our findings and current evidence, we advocate for the following improvements in ED management of incidentalomas:

- structured radiology reporting, including a “Recommendations” section referencing criteria of the American College of Radiology (ACR), the European Society of Endocrinology (ESE) and the Fleischner Society guidelines;
- automated follow-up alerts to general practitioners or specialty clinics for lesions requiring surveillance;
- visual clinical algorithms, integrated into ED radiology software systems, to assist non-specialist clinicians in triage and follow-up planning;
- establishment of a clinical incidentaloma registry within the ED setting to support quality-improvement initiatives and future research.

We further advocate for the integration of international guidelines and evaluation of overdiagnosis risk. To enhance the clinical applicability of our findings, we retrospectively evaluated the management of incidental neoplastic findings against established international guidelines of the ACR Incidental Findings Committee. The ACR provides detailed management recommendations for incidental findings in major anatomical regions (e.g., adrenal glands, liver, kidneys, lungs), stratified by radiologic features, such as size, margins, and enhancement, and clinical risk factors.^9^ The 2017 Fleischner criteria provide evidence-based algorithms for the management of incidental pulmonary nodules detected on CT, with recommendations based on nodule size, morphology, and patient risk profile (e.g., age, smoking history).^12^ For adrenal incidentalomas, the ESE recommends workup based on size (> 4 cm), attenuation values (< 10 Hounsfield units), contrast wash-out characteristics, and functional status.^12^

All detected neoplastic incidentalomas were retrospectively reviewed and, where data allowed, classified in accordance with these clinical frameworks. In particular, we compared CT-based lesions in the lungs, adrenal glands, and abdomen with guideline-directed thresholds for further imaging, biopsy, or follow-up. We also noted the presence or absence of explicit radiological recommendations and whether follow-up plans were communicated to patients at ED discharge.

### Assessment of Overdiagnosis and the Cascade Effect

The unintended discovery of potentially oncologic lesions can initiate a chain of diagnostic and therapeutic interventions, often termed the “cascade effect.” This may include the following: unnecessary or low-value procedures, particularly in elderly or frail patients; invasive diagnostic interventions (e.g., biopsies, surgical exploration) with associated iatrogenic risk; increased psychological distress, including health anxiety triggered by ambiguous or unexpected results; and healthcare system burden, with resource-intensive follow-up often lacking standardized coordination. In our cohort 72% of patients with incidentalomas were discharged directly from the ED, and documented follow-up plans were absent from most discharge summaries. Based on guideline thresholds, we estimate that 15–20% of cases could represent potential overdiagnosis, consistent with prior systematic reviews.^2^,^8^ While direct assessment of psychological impact was beyond the scope of this study, future research should consider validated tools such as the Hospital Anxiety and Depression Scale (to quantify emotional consequences following incidental cancer findings).^13^,^14^

**Figure 2:**
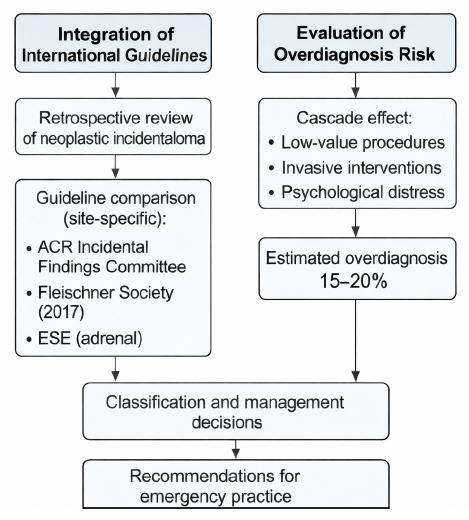
Methodological framework for guideline integration and overdiagnosis risk evaluation in a study of emergency department incidentalomas. *ACR*, American College of Radiology; *ESE*, European Society of Endocrinology.

In Figure 2 we present the guideline alignment process, in which neoplastic incidentalomas were retrospectively classified and compared with site-specific recommendations from the ACR, Fleischner Society, and European Society of Endocrinology. On the right, we outline the assessment of overdiagnosis risk, focusing on potential “cascade effects” such as unnecessary procedures, psychological burden, and resource overuse.

## LIMITATIONS

This study has several limitations. It is limited by its retrospective, single-center design, which may reduce generalizability. Secondly, we did not assess the psychological impact of incidental findings. Additionally, we did not formally separate incidentalomas from newly diagnosed malignancies, potentially related to the reason for ED presentation. While both were reported together, this may have influenced prevalence estimates and clinical interpretation. Future studies should attempt to differentiate these categories more explicitly, as their management pathways and prognostic implications differ. Data were based on discharge summaries, which may have under-reported clinical intent. Finally, we did not perform a formal sample-size calculation or power analysis, as the study design was retrospective and descriptive in nature. This limitation is inherent to the observational approach and should be considered when interpreting subgroup comparisons.

## CONCLUSION

Detection of neoplastic incidentalomas is relatively frequent in emergency settings, particularly among elderly patients. Based on the results of this study, we advocate for increased awareness among emergency physicians regarding patients presenting with minor emergency complaints. These findings underscore the need for guideline-informed management of incidentalomas. Future studies are required to better investigate this issue.

## Figures and Tables

**Table 1:** Demographic and clinical characteristics of patients with incidentalomas in a study of the prevalence of incidentalomas identified in the emergency department.

Age Group	Count (n)	Percentage (%)	Female (n)	Male (n)	Mean Age (±SD)
18–40 years	4	7.5%	3	1	32.5 (±5.1)
40–65	16	30.2%	10	6	52.4 (±6.3)
> 65	33	62.3%	24	9	73.8 (±5.7)
Total	53	100%	37	16	—

**Table 2 t2-wjem-27-842:** Triage codes, imaging modalities, and prevalence of incidentaloma findings on imaging in the emergency department.

Triage Code	Number of Cases	Percentage (%)	Most Common Imaging	Estimated Prevalence (per 1,000 ED visits)
Green*	33	62%	CT	2.3
Blue**	20	38%	Ultrasound / Radiograph	0.9
**Total**	**53**	**100%**	—	**1.52**

* Green = not urgent.

** Blue = deferred urgency.

**Table 3 t3-wjem-27-842:** Presenting symptoms by tumor site in a study of emergency department findings of incidentalomas.

Tumor site	Most common symptom	Frequency of association
Gastrointestinal	Abdominal pain	82%
Pulmonary	Dyspnea / fatigue	61%
Breast	Trauma / dizziness	100% (female-only cases)
Ovarian	Abdominal bloating	60%
Cerebral	Seizures	75%
Renal	Anemia / hematuria	50%
